# Comparison of Media for the Detection of *Campylobacter jejuni* Using a Commercial RT-PCR System

**DOI:** 10.3390/pathogens14020166

**Published:** 2025-02-08

**Authors:** Elena G. Olson, Aaron R. Bodie, Haley A. Tarcin, Peter M. Rubinelli, Savannah F. Applegate, Tyler P. Stephens, Michael J. Rothrock, Steven C. Ricke

**Affiliations:** 1 Meat Science and Animal Biologics Discovery Program, Department of Animal and Dairy Sciences, University of Wisconsin, Madison, WI 53706, USA; 2 Poultry Science Department, University of Georgia, 120 D W Brooks Dr., Athens, GA 30602, USA; 3 Department of Food Science, University of Arkansas, 2650 Young Avenue, Fayetteville, AR 72704, USA; 4 Hygiena, 941 Avenida Acaso, Camarillo, CA 93012, USA; 5 Micro Enviro Tech LLC, 233 Twin Oaks Dr., La Vernia, TX 78121, USA; 6 United States Department of Agriculture, Agricultural Research Service, Athens, GA 30605, USA

**Keywords:** *Campylobacter jejuni*, RT-PCR, detection, Mueller–Hinton, enrichment

## Abstract

The accurate quantification of *Campylobacter jejuni* in poultry samples is critical for ensuring food safety and compliance with regulatory standards. This study evaluated the performance of three enrichment media—Mueller–Hinton Broth (MHB), Bolton’s Blood-Free Broth 2x (BFBB2x), and Buffered Peptone Water (BPW)—in supporting *C. jejuni* detection and quantification using the BAX^®^ Q7-RT PCR system and traditional plate count methods. Results demonstrated high reliability across all media types, with BFBB2x and MHB showing the strongest correlations (R^2^ = 0.99) for the BAX^®^ system. BFBB2x exhibited the lowest RMSE (0.13), while MHB balanced precision (RMSE = 0.4) with sensitivity. For plate counts, MHB and BPW achieved the highest correlations (R^2^ = 0.99) and precision (RMSE = 0.26), with MHB demonstrating the lowest detection limit (2.56 log_10_ CFU/mL) compared to BFBB2x (2.93 log_10_ CFU/mL) and BPW (3.31 log_10_ CFU/mL). The findings underscore MHB’s robustness as an enrichment medium, offering consistent performance across both molecular and culture-based methods. The current study supports MHB as the more effective medium for the reliable and precise quantification of *C. jejuni* in poultry-associated matrices, highlighting its utility in minimizing contamination risks and enhancing food safety. Future research should explore its applicability in diverse poultry products and production environments.

## 1. Introduction

*Campylobacter jejuni* is a conspicuous foodborne pathogen and a worldwide leading cause of human illness [1,2]. In addition to causing gastrointestinal symptoms, it can lead to severe post-infection complications such as Guillain–Barré syndrome and reactive arthritis [1]. Poultry meat products are a primary source of *C. jejuni* infections, as chickens naturally harbor the bacterium in their intestinal tract, and it coexists with the indigenous microbiota [3,4]. *C. jejuni* is a microaerophilic organism requiring low oxygen levels, carbon dioxide, and hydrogen for growth [1,3]. Its optimum growth temperature, 42 °C, aligns closely with the body temperature of poultry, facilitating colonization [3]. Morphologically, *C. jejuni* is a Gram-stain negative, curved, or spiral-shaped bacterium exhibiting corkscrew motility. Under stressful environmental conditions, it can shift to coccoid or filamentous forms, becoming viable but difficult to cultivate [5].

Given the significance of *C. jejuni* as a foodborne pathogen, the reliable detection and quantitation of contaminated food products are essential. Numerous selective and enrichment media have been developed to isolate and promote the growth of *Campylobacter* species. Among these, Bolton’s Blood-Free Broth is widely used by government regulatory agencies such as the United Sates Department of Agriculture—Food Safety and Inspection Service (USDA-FSIS) due to its effective formulation, which includes yeast extract, α-ketoglutaric acid, sodium pyruvate, sodium metabisulphite, and hemin; ingredients that support the specific nutritional needs of *Campylobacter* [6,7,8,9]. Similarly, Mueller–Hinton Broth, containing beef extract, acid hydrolysate of casein, and starch, provides essential nutrients and absorbs toxic metabolites, creating a suitable growth environment [10].

In addition to culture-based methods, molecular techniques such as polymerase chain reaction (PCR) have been increasingly utilized to detect and quantify *C. jejuni*. Effective enrichment is critical to achieving detection thresholds, enhancing PCR consistency, and minimizing PCR inhibition [11]. The BAX^®^ System Real-Time PCR assay is a rapid and specific molecular method for detecting and quantifying *Campylobacter* in food and environmental samples, approved by AOAC International. It targets a 400-base-pair species-specific region and specifically detects *C. jejuni*, *C. lari*, and *C. coli* without cross-reacting with other bacteria commonly found in poultry samples, offering advantages over phenotypic and antibody-based methods [12]. Recent studies, such as Bodie et al. (2024), have demonstrated the utility of the BAX^®^ PCR system for detecting and semi-quantitating *Campylobacter* species, using Bolton’s Blood-Free Broth as the pre-enrichment medium [12]. The current study compared the performance of Bolton’s Blood-Free Broth, Mueller–Hinton Broth, and Buffered Peptone Water as enrichment media for *C. jejuni* before PCR detection. We evaluated these media to determine their relative effectiveness in supporting *C. jejuni* growth and facilitating reliable PCR-based detection.

## 2. Methods and Materials

### 2.1. Bacterial Strain and Culture Conditions

*Campylobacter* subsp. *jejuni* ATCC 700819 was used in the current study. *C. jejuni* was stored at −80 °C in Tryptic Soy Broth (Rochester, NY, USA) containing 20% (*v*/*v*) glycerol. A frozen stock of *C. jejuni* was streak-plated from the −80 °C vials on Modified Charcoal–Cefoperazone–Deoxycholate Agar (mCCDA; Himedia, Mumbai, India) and incubated under microaerophilic conditions (5% O_2_, 10% CO_2_, and 85% N_2_) at 42 °C for 24 h. Next, a single colony of *C. jejuni* was inoculated into 20mL of either Mueller–Hinton Broth (MHB; Neogen, Lansing, MI, USA), 2x Bolton’s Blood-Free Broth (BFBB2x; Neogen, Lansing, MI, USA), or Buffered Peptone Water (BPW; ThermoScientific, Waltham, MA, USA). The cultures were incubated under microaerophilic conditions at 42 °C with constant shaking at 100 revolutions per minute (RPM) for 24 h.

### 2.2. Detection Curves and Detection Limits of Campylobacter in Pure Cultures by BAX^®^ Q7-RT System

**Figure 1:**
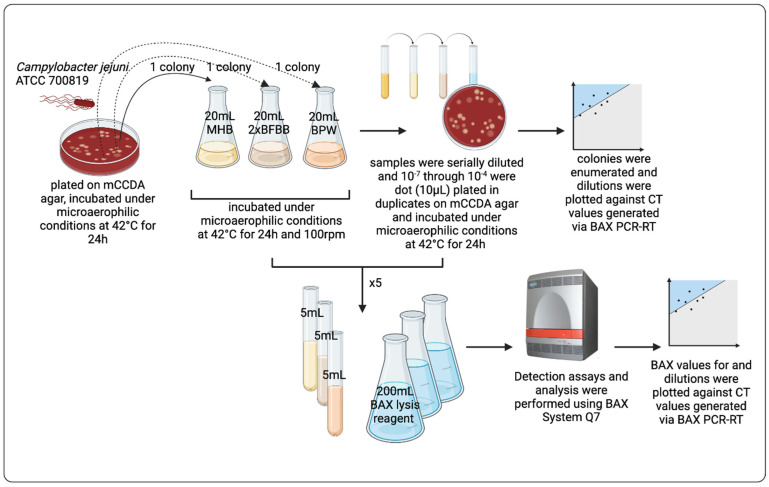
Overview of the experimental design in the current study. Created in BioRender, https://BioRender.com/q11g054. (accessed on 6 February 2025).

The detection limits of the BAX^®^ System were assessed from pure cultures of the *Campylobacter* grown in three different media. After the cultures were grown in three different media, samples were 10-fold serially diluted, and 10^−7^ to 10^−4^ dilutions were dot-plated (10 μL) in duplicates on mCCDA plates, incubated under microaerophilic conditions at 42 °C for 24 h, and enumerated the next day. All original and diluted samples were quantified via BAX^®^ Q7-RT PCR (KIT2018, Hygiena, Camarillo, CA, USA) using the *Campylobacter* species assays (following the manufacturer’s instructions). Briefly, 5 mL of each sample was added to cluster tubes with 200 mL of prepared BAX^®^ System lysis reagent. Lysis was performed by heating the tubes for 20 min at 37 °C and 10 min at 95 °C and then cooling the tubes at 4 °C for at least 5 min. Lysate was used to hydrate a PCR tablet for the BAX^®^ System assays. PCR tubes were loaded into the BAX^®^ System Q7 instrument and run according to the procedure described in the BAX^®^ System user guide. All samples were examined in five replicates. The results were analyzed with BAX^®^ Q7 software (version 2.8). Detection curves of the BAX^®^ RT systems were generated based on the PCR threshold cycle (CT) values. Phenotypical characteristics confirmed presumptive positive *Campylobacter* colonies. This study was performed on three separate timely independent trials. The overview of the experimental design is described in Figure 1.

### 2.3. Statistical Analysis

Each dilution series of media was statistically analyzed using a linear regression analysis in R Studio [13], allowing an association between dilution series and *C. jejuni* detection in the BAX^®^ Q7 PCR. An analysis of variance (ANOVA) was used to determine whether there were any significant differences in the detection limits of different media in the BAX^®^ Q7 PCR. Tukey’s Honestly Significant Difference (HSD) was utilized to determine pairwise differences between the media, with significance determined at *p* < 0.05. Tukey’s HSD test was used as it controls for Type I error while allowing for comparisons between all group means. The assumptions of normality (Shapiro–Wilk test, *p* > 0.05) and homogeneity of variances (Levene’s test, *p* > 0.05) were met, justifying the use of this post hoc method [14,15].

## 3. Results

### 3.1. Development and Evaluation of Linear-Fit Equations for the Quantification of Campylobacter

The CT values generated by the BAX^®^ Q7 system at the 24-h timepoint were analyzed to develop linear-fit equations, along with their corresponding R^2^ values and Root Mean Square Errors (RMSE). These linear equations were used to estimate pre-enriched *Campylobacter* levels, simulating the logarithmic growth phase of the bacteria. The R^2^ value denotes the proportion of variation in the dependent variable described by the linear model, highlighting the relationship between CT value variation, enrichment time, and known bacterial inoculation levels. RMSE quantifies the standard deviation of the residuals, indicating how well the CT values predict the actual *C. jejuni* concentrations. Each linear equation was evaluated based on specific statistical criteria: R^2^ > 0.80, log RMSE < 0.60, and an enumerable range of 4.00 to 8.00 log_10_ CFU/mL. Equations that did not meet these parameters resulted in inaccurate estimations compared to known spike levels. The specified statistical criteria and an enumerable range are standard benchmarks for evaluating the performance of linear regression models in microbiological quantification studies. These thresholds ensure that the model explains a substantial portion of the variance (R^2^ > 0.80), maintains low prediction errors (log RMSE < 0.60), and operates effectively within the relevant concentration range (4.00 to 8.00 log_10_ CFU/mL) [16]. This underscores the importance of adhering to these statistical benchmarks while developing and validating enrichment protocols and linear fit equations. Ensuring compliance with these criteria is crucial for creating a reliable and rapid quantification tool.

### 3.2. Detection Limits Within Each Media Type

**Figure 2:**
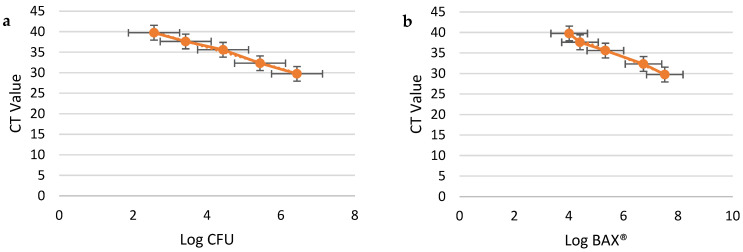
*Campylobacter jejuni* dilution samples in Mueller–Hinton Broth. X-axis: Log CFU/mL plate count; Y-axis: CT values from BAX^®^ RT-PCR (**a**). X-axis: Log CFU/mL from BAX^®^ RT-PCR; Y-axis: CT values from BAX^®^ RT-PCR (**b**). Data were evaluated through a linear-fit equation applied to Log CFU and CT values (y = −2.5971x + 46.583, R^2^ = 0.9946, and RMSE = 0.2634) (**a**) and Log BAX^®^ and CT values (y = −2.6677x + 49.949, R^2^ = 0.9884, and RMSE = 0.3861) (**b**).

**Figure 3:**
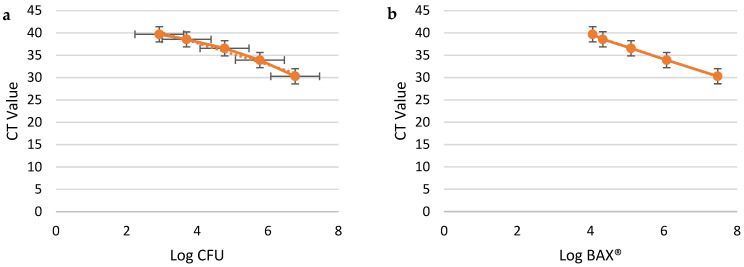
*Campylobacter jejuni* dilution samples in Bolton Broth. X-axis: Log CFU/mL plate count; Y-axis: CT values from BAX^®^ RT-PCR (**a**). X-axis: Log CFU/mL from BAX^®^ RT-PCR; Y-axis: CT values from BAX^®^ RT-PCR (**b**). Data were evaluated through a linear-fit equation applied to Log CFU and CT values (y = −2.4082x + 47.342, R^2^ = 0.9712, and RMSE = 0.5738) (**a**) and Log BAX^®^ and CT values (y = −2.71x + 50.458, R^2^ = 0.9984, and RMSE = 0.1346) (**b**).

**Figure 4:**
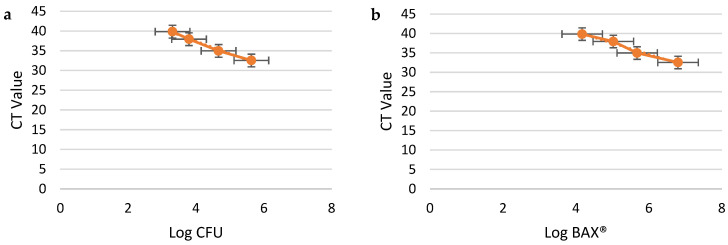
*Campylobacter jejuni* dilution samples in Buffered Peptone Water. X-axis: Log CFU/mL plate count; Y-axis: CT values from BAX^®^ RT-PCR (**a**). X-axis: Log CFU/mL from BAX^®^ RT-PCR; Y-axis: CT values from BAX^®^ RT-PCR (**b**). Data were evaluated through a linear-fit equation applied to Log CFU and CT values (y = −3.1342x + 49.96, R^2^ = 0.9913, and RMSE = 0.2598) (**a**) and Log BAX^®^ and CT values (y = −2.8764x + 51.907, R^2^ = 0.9793, and RMSE = 0.4013) (**b**). Dilution 4 was omitted from graphs as the CT value, Log CFU, and Log BAX^®^ were all zero.

The linear regression analysis demonstrated strong correlations between the log CFU/mL values from plate counts and the CT values generated by the BAX^®^ Q7-RT PCR system across all three media types (Figure 2a, Figure 3a and Figure 4a). Both methods demonstrated high reliability in quantifying *Campylobacter jejuni* concentrations, as evidenced by their high coefficients of determination (R^2^) and low RMSE values. Both BAX^®^ RT-PCR and plate count methods demonstrated high reliability in quantifying *C. jejuni*, with results varying slightly across the tested enrichment media. For the BAX^®^ system, MHB and BFBB2x exhibited the strongest correlations (R^2^ = 0.99), but BFBB2x had a lower RMSE (0.13) compared to MHB (0.4), indicating higher precision. Buffered BPW showed slightly lower correlation (R^2^ = 0.97) and a higher RMSE (0.4), reflecting reduced accuracy in this system. For plate counts, MHB and BPW displayed equally strong correlations (R^2^ = 0.99) with identical RMSE values (0.26), whereas BFBB2x had a slightly lower R^2^ (0.97) and higher RMSE (0.57), indicating less precision. Plate counts further validated MHB’s superior performance, showing the lowest detection limit (2.56 log_10_ CFU/mL), compared to BFBB2x (2.93 log_10_ CFU/mL) and BPW (3.31 log_10_ CFU/mL) (Table 1). These findings highlight MHB as the most effective enrichment medium, offering a balanced combination of precision and sensitivity for both BAX^®^ RT-PCR and plate count methods, making it the preferred choice for reliable and accurate *C. jejuni* quantification.

Enumerable ranges varied between media types, as shown in Table 1 and Table 2 and Figure 2a,b, Figure 3a,b and Figure 4a,b. BAX^®^ RT-PCR estimates yielded broader enumerable ranges (MHB: 4.01–7.52 log_10_ CFU/mL; BFBB2x: 4.05–7.46 log_10_ CFU/mL; BPW: 4.17–6.79 log_10_ CFU/mL) compared to plate counts (MHB: 2.56–6.43 log_10_ CFU/mL; BFBB2x: 2.92–6.77 log_10_ CFU/mL; BPW: 3.31–5.63 log_10_ CFU/mL). The standard curves based on BAX^®^ RT-PCR estimates exhibited a more extensive enumerable range than those derived from plate count estimates. However, no significant differences were observed when standard curve slopes were compared across media.

The detection limits, defined as the lowest concentration reliably detected, varied across the three media types. The RMSE values were lowest in MHB and highest in BPW, reflecting greater variability in the latter’s CT values at low bacterial concentrations. This variation is visually represented in Figure 2b, Figure 3b and Figure 4b, which depict CT values from BAX^®^ RT-PCR as a function of the estimated bacterial concentrations: MHB (Figure 2b) had the highest sensitivity, with CT values accurately predicting bacterial levels across a broader range. BFBB2x (Figure 3b) demonstrated intermediate sensitivity and precision. BPW (Figure 3b) exhibited greater variability in detection limits. The R^2^ values, although consistently high across media, also suggest that the detection precision is highest in MHB and lowest in BPW. The differences in detection limits across media types highlight the influence of media composition on the performance of the BAX^®^ Q7-RT PCR system.

These results highlight the utility of BAX^®^ RT-PCR for rapid and high-throughput detection, with plate counts providing complementary validation. Statistical analysis confirmed significant relationships between bacterial concentrations and CT values, with the ANOVA test revealing differences in detection limits among media (*p* < 0.05). Post hoc comparisons indicated that MHB provided the highest sensitivity and precision, followed by BFBB2x and BPW.

## 4. Discussion

The current study’s results provide critical insights into the performance of the BAX^®^ Q7-RT PCR system for detecting *C. jejuni* across different enrichment media, with direct implications for poultry production and food safety. A potential limitation of this study is the variability in bacterial load among individual colonies, which may contribute to differences in detection sensitivity across media. A standardized bacterial suspension for inoculation could enhance consistency and comparability, reducing variation due to colony size differences. Despite this, the observed replicates confirmed that the method was effective, demonstrating reproducibility across media types. Future studies could incorporate this refinement to improve precision in comparative assessments [17,18].

Detection limits and enumerable ranges varied significantly among Mueller–Hinton Broth (MHB), Bolton’s Blood-Free Broth (BFBB2x), and Buffered Peptone Water (BPW), highlighting the impact of media composition on assay sensitivity and precision. The lowest detection limit observed with MHB (2.56 log_10_ CFU/mL) suggests the capability of the media to detect low concentrations of *C. jejuni* compared to other media used in the current study. Real-time PCR assays typically detect multiple gene targets, with detection limits influenced by various factors, including DNA extraction efficiency, sample matrix complexity, and potential PCR inhibitors [19,20]. The observed LOD of 2.56 log_10_ CFU/mL for MHB is slightly higher than expected for RT-PCR detection. This discrepancy may be attributed to the presence of background microbiota in poultry samples, which can affect DNA recovery and amplification efficiency [21]. Additionally, variations in LOD across different studies can result from differences in sample preparation, DNA extraction protocols, and PCR assay conditions [22]. Comparatively, previous studies have reported LOD values ranging from approximately 1.0 to 3.0 log_10_ CFU/mL for *Campylobacter* detection in food and environmental samples using RT-PCR [19,23]. While some studies have achieved lower LODs using enrichment steps prior to PCR [24], direct detection without enrichment often results in slightly higher detection limits, as seen in our study. Culture-based methods typically demonstrate LODs in a similar range, but their extended turnaround time and susceptibility to competing microbiota make RT-PCR a preferable alternative for rapid detection [21].

Utilizing MHB over standard enrichment media such as Bolton Broth may provide a valuable tool for early and accurate identification in poultry rinsates. Poultry rinsate, a commonly used sample type in regulatory and industry testing, often contains variable bacterial loads due to carcass handling, processing practices, and storage conditions. Moreover, the ability of MHB to provide a broader enumerable range and outperform BFBB2x (2.56 to 6.44 log_10_ CFU/mL via plate counts and 4.01 to 7.52 log_10_ CFU/mL via BAX^®^) enhances its utility for quantifying *C. jejuni* at both low and high contamination levels, crucial for monitoring compliance with performance standards and identifying critical control points in processing facilities [15]. In contrast, BPW demonstrated a higher detection limit (3.31 log_10_ CFU/mL) and a narrower enumerable range (3.31 to 5.6 log_10_ CFU/mL via plate counts and 4.17 to 6.79 log_10_ CFU/mL via BAX^®^). While BPW is commonly used in microbiological testing due to its buffering capacity, its reduced sensitivity may limit its applicability in scenarios requiring the detection of low bacterial concentrations, such as during pre-harvest interventions or in rinse samples from carcasses subjected to enhanced decontamination treatments [17].

The broader enumerable ranges observed with BAX^®^ RT-PCR estimates compared to plate counts highlight the potential of this molecular method for high-throughput screening in poultry production. The BAX^®^ system’s rapid detection and quantification capabilities can support proactive measures to control *Campylobacter*, reducing contamination risks during processing and improving product safety [18]. Furthermore, the absence of significant differences in standard curve slopes among the media types suggests that while media choice affects detection limits and ranges, the BAX^®^ system maintains robust linearity across different enrichment conditions, ensuring consistent quantification. From a production perspective, these findings underscore the importance of media selection when optimizing detection protocols for *Campylobacter* in poultry. Based on the highest sensitivity and broader range, MHB may be particularly advantageous for detecting contamination in critical areas such as post-chill carcass rinses, where bacterial loads are typically low [19]. Conversely, BFBB2x and BPW may serve as complementary media for applications where the rapid growth and recovery of stressed *Campylobacter* cells are prioritized [20].

The BAX^®^ Q7-RT PCR system, when paired with suitable enrichment media, offers faster and more efficient detection and quantification of *Campylobacter jejuni* in poultry samples compared to traditional plating methods. While plating requires 48 to 72 h for visible colonies to develop, the BAX^®^ system can deliver results within 24 to 30 h, including the enrichment step, making it a faster alternative [12]. Additionally, it provides higher sensitivity and specificity, allowing detection in complex microbial environments and at low concentrations, with reduced variability compared to manual colony counting [11]. Moreover, the system allows for quantitation through real-time PCR, enhancing its utility in risk assessment and microbial load studies, where plating is primarily qualitative or semi-quantitative. However, the BAX^®^ system does not recover viable isolates, a critical advantage of traditional plating, essential for regulatory compliance and source tracking in food safety investigations. This limitation highlights the complementary roles of both methods in minimizing contamination, ensuring food safety, and protecting public health. Future research should focus on validating the BAX^®^ system across diverse poultry matrices, such as carcass rinses and processed meat, to confirm its applicability and reliability at various stages of production and processing. Matrix composition, PCR inhibitors, and microbial background can influence the detection sensitivity of *Campylobacter* species in complex food products [21,23]. Expanding validation across different matrices would enhance the method’s generalizability for food safety applications. Beyond analytical performance, practical implementation in food safety laboratories depends on cost, ease of use, and regulatory acceptance. Compared to culture-based methods, which require 48 to 72 h, the BAX^®^ Q7 system provides results within 24 h, reducing processing time and minimizing false positives [24]. Although PCR-based assays require specialized equipment and trained personnel, potentially increasing costs, automation and high-throughput capabilities can offset expenses by improving efficiency [23,25]. Although molecular methods are increasingly preferred for rapid detection, culture-based techniques remain essential for confirmation and antimicrobial susceptibility testing [22]. A combined approach integrating PCR with enrichment and culture methods can offer a more comprehensive solution for food safety monitoring. Further studies on cost-effectiveness and real-world application would support the broader adoption of the BAX^®^ Q7 system.

## 5. Conclusions

The current study provides valuable insights into the performance of the BAX^®^ Q7-RT PCR system for detecting *Campylobacter jejuni* across different enrichment media, with direct implications for poultry production and food safety monitoring. The findings highlight the impact of media selection on detection sensitivity, with Mueller–Hinton Broth (MHB) demonstrating the lowest detection limit and the broadest enumerable range, making it a promising choice for quantifying *C. jejuni* at both low and high contamination levels. Comparatively, Bolton’s Blood-Free Broth (BFBB2x) and Buffered Peptone Water (BPW) showed higher detection limits and narrower enumerable ranges, suggesting their suitability in specific applications where rapid growth or stress recovery is prioritized. The BAX^®^ system provides a faster and more sensitive alternative to culture-based methods, though its inability to recover viable isolates underscores the need for complementary approaches. Standardizing bacterial suspensions and validating the method across diverse poultry matrices will further enhance its reliability. Future work should also assess cost-effectiveness and regulatory acceptance to facilitate broader adoption in food safety laboratories.

## Figures and Tables

**Table 1:** Average bacterial counts (Log CFU/mL) from modified Charcoal Cefoperazone Deoxycholate Agar (*mCCDA*) plates and corresponding cycle threshold (CT) values for *Campylobacter jejuni* grown in Mueller–Hinton Broth (MHB), Blood-Free Bolton Broth (BFBB2x), and Buffered Peptone Water (BPW). Values represent the means of three independent trials, recorded at serial dilution levels from 0 to 4.

LogCFU	0	1	2	3	4
MHB	6.44	5.44	4.44	3.42	2.56
BFBB2x	6.78	5.78	4.78	3.70	2.93
BPW	5.63	4.67	3.80	3.31	0.00
CT	0	1	2	3	4
MHB	29.74	32.32	35.59	37.61	39.75
BFBB2x	30.30	33.92	36.56	38.57	39.69
BPW	32.54	34.97	37.94	39.84	0.00

**Table 2:** Average Log BAX values and corresponding cycle threshold (CT) values for *Campylobacter jejuni* grown in Mueller–Hinton Broth (MHB), Blood-Free Bolton Broth (BFBB2x), and Buffered Peptone Water (BPW). Values represent the means of three independent trials, recorded at serial dilution levels from 0 to 4.

LogBAX	0	1	2	3	4
MHB	7.52	6.73	5.34	4.41	4.01
BFBB2x	7.47	6.08	5.10	4.33	4.05
BPW	6.80	5.68	5.02	4.17	0.00
CT	0	1	2	3	4
MHB	29.74	32.32	35.59	37.61	39.75
BFBB2x	30.30	33.92	36.56	38.57	39.69
BPW	32.54	34.97	37.94	39.84	0.00

## Data Availability

The data presented in this study are available on request from the corresponding author. The data are not publicly available due to the proprietary nature of the research.
